# Credit contingent interest rate swap pricing

**DOI:** 10.1186/s40929-017-0015-x

**Published:** 2017-10-03

**Authors:** Haohan Huang, Huaxiong Huang, Eugene Wang, Hongmei Zhu

**Affiliations:** 1 RBC Financial Group, 222 Bay St, Toronto, M5K 1G8 ON Canada; 2 Department of Mathematics and Statistics, York University, 4700 Keele Street, Toronto, ON, M3J 1P3 Canada; 3 Fields Institute, 222 College Street, Toronto, ON, M5T 3J1 Canada

**Keywords:** Credit value adjustment (CVA), Credit contingent interest rate swap (CCIRS), Counter-party risk, Partial differential equations

## Abstract

Credit value adjustment (CVA) is an adjustment to an existing trading price based on the counterparty-risk premium. Currently, CVA is computed with an implicit assumption that the replacement contract is default-free after the original counterparty defaults, with the assumption that those trades will not re-assigned. In the actual counterparty default settlement, it is the norm that trades will be re-assigned, especially on the buy side. Since the counterparty of the replacement contract could also default within the lifetime of an existing contract, ignoring the possibility of counterparty defaults of replacement contracts will either under or over estimate the cost of the risk. An important practical question is, therefore, how to estimate under/over pricing of CVA under current practice.

In this paper, we considered the pricing of credit contingent interest rate swap (CCIRS) or credit contingent default swap (CCDS), which is considered the CVA hedge for interest rate swaps (IRS). We derived partial differential Eqs. (PDEs) satisfied by the approximated CVA with the assumption that the replacement contracts do not default. For comparison purposes, we also derived the PDEs for the cost of CVA by relaxing the assumption of default-free replacement contracts with a finite number of counterparty defaults. It shows that the no-default and two default cases can be derived within the same analytical solution framework, similar to the Funding Valuation Adjustment (FVA) problem where continuous funding is a reasonable assumption. The finite number of default case is non-trivial. The PDE for the two default case is derived in this paper.

We calibrate our model based on market data and carry out extensive computations for the purpose of comparing these three CVAs. Our basic finding is that the values of the two CVAs are close for top rated counterparties. On the other hand, for counterparties with lower credit ratings, the difference among the two CVAs can be significant.

## Introduction

Counterparty Credit Valuation Adjustment(CVA) is defined as an adjustment to the price when counterparty risk is considered. In recent years, CVA and other valuation adjustments such as Debt Valuation Adjustment (DVA), Funding Valuation Adjustment (FVA), Capital Valuation Adjustment (KVA),and Margin Valuation Adjustment (MVA), have been driving credit trading and counterparty credit risk, funding and capital cost management of financial institutes. As an overhaul of financial reform to address the financial crisis, counterparty risk related regulations such as counterparty credit risk (CCR)/CVA regulatory capital, central clearing, and initial margin have been applied by the regulators, fundamentally changing the financial risk management landscape of the financial industry.

For an over-the-counter (OTC) derivative, the counterparty risk always exists. In some of the earlier pricing literature for credit swaps, counterparty and investor are considered to be default free as in Duffie [1] and Hull et al. [2]. Counterparty default risk is considered in some studies, cf. Hull et al. [3] while the volatility of the credit spread is neglected (the hazard rate is assumed to be a constant). In other studies, volatility of the credit spread is included but the interest rate of the underlying asset is assumed to be a constant as in Brigo et al. [4] and Sorensen [5]. In more recent work Brigo et al. [6–8], both stochastic interest rate and hazard rate models are used. In Brigo et al. [6, 8], both investor and counterparty defaults, or “bilateral counterparty risk", are included in the models. However, a possible correlation between credit spread volatility and interest rate is not considered. In Assefa et al. [9], Crepey [10] and Crepey [11], applications of bilateral counterparty risk have been discussed and analyzed.

Currently, CVA is usually computed with an implicit assumption that the contract will stop and exposure at default is computed without counterparty risk considered. This assumes that the exposure is computed with a default free assumption. However, in the actual counterparty default settlement, it is the norm that trades will be re-assigned, especially on the buy side. Even though CVA will be computed when the trade is actually re-assigned and managed as the new counterparty exposure, the implication of trade re-assignment is not considered when the initial contract is signed. A few studies have covered a substitution closeout which has taken into account the risk of default of the survived party in bilateral CVA calculation, such as in Brigo et al. [12] and also Brigo et al. [13]. On the other hand, in the FVA calculation it is the norm to assume that the funding cost of the exposure is considered in the exposure calculation as in Anderson et al. [14], Burgard et al. [15] and Piterbarg [16].

In this paper, we considered the pricing of credit contingent interest rate swap (CCIRS) or credit contingent default swap (CCDS). When the reference entity defaults, the CCIRS has the right to settle into the underlying swap. It serves as the hedging trade of counterparty risk and valued very similarly to the CVA of an IRS. Following recent literature, we assume that both the hazard rate and the interest rate are stochastic with a possible correlation for tractability. Our main objective is to derive partial differential equations based model and investigate the effect of a possible second default of the replaced counterparty, which have been neglected in the existing literature. The basic question we addressed was whether it is justified to ignore the cost associated with defaultable replacement contract of the original CCIRS under normal market conditions. To do so, we first solved the pricing problem of CCIRS without the possibility of a second default. We also find the price of a CVA by allowing a second default, and compare the prices using reasonable parameter values for the interest rate and the hazard rate. For simplicity, we restrict the number of defaults to two in this paper and the more general case will be considered in a follow up paper.

The rest of this paper is arranged as follows. In “Credit contingent interest rate swap” section, we establish that the pricing problem of CCIRS is equivalent to a CVA problem for interest rate swap. In “CCIRS with default-free replacement contracts” section, the pricing problem for CCIRS is formulated with the assumption of a default-free replacement contract. In “CCIRS with defaultable replacement contract” section, pricing of a CCIRS that allows a second default is considered and comparisons with the one-default price is given. Numerical results are given in “Numerical results” section. We conclude this paper in “Conclusion” section with a discussion of the limitations of our method and possible directions for future research.

## Credit contingent interest rate swap

An interest-rate swap is a contract between two parties where one party (e.g. the bank) receives a fixed amount periodically in exchange for the London Interbank Offered Rate (LIBOR) linked floating payments to the counterparty. When a counterparty defaults, a replacement contract is established and there is a probability that the cost of the replacement contract is significantly higher than that of the original one.

Its credit value adjustment (CVA) is the expected cost due to interest rate changes as well as the replacement costs in the cases of defaults of both parties. In Brigo et al. [6, 8], a general formula for pricing CVA was introduced using the following notations:

$$\begin{array}{*{20}l} \tau_{I}&:\text{default time of investor,}\\ \tau_{C}&: \text{default time of counterparty,}\\ T&: \text{maturity of the underlying,}\\ A&=\{\tau_{I}\leq \tau_{C}\leq T\},\qquad B=\{\tau_{I}\leq T\leq \tau_{C}\},\\ C&=\{\tau_{C}\leq \tau_{I}\leq T\},\qquad D=\{\tau_{C}\leq T\leq \tau_{I}\}, \\ E&=\{T\leq \tau_{I}\leq \tau_{C}\},\qquad F=\{T\leq \tau_{C}\leq \tau_{I}\}, \\ \Pi(t,T)&: \text{default - free trade value at time~} t \text{~with maturity~} T,\\ \Pi^{D}(t,T)&: \text{trade value after adjustment at time~} t \text{~with maturity~} T,\\ {LGD}_{I}&: \text{loss given default ratio of investor}, \\ {LGD}_{C}&: \text{loss given default ratio of counterparty}, \end{array} $$

The new price with CVA under these notations is given as:

$$\begin{array}{*{20}l} E\left\{\Pi^{D}(t,T)|\mathfrak{F}_{t}\right\}&=E\{\Pi(t,T)|\mathfrak{F}_{t}\}\\ &\quad+E\{\text{LGD}_{I}\cdot I(A\cup B)\cdot P(t,\tau_{I})\cdot\left[-\text{NPV}(\tau_{I})\right]^{+}|\mathfrak{F}_{t}\}\\ &\quad-E\{\text{LGD}_{C}\cdot I(C\cup D)\cdot P(t,\tau_{C})\cdot\left[\text{NPV}(\tau_{C})\right]^{+}|\mathfrak{F}_{t}\}, \end{array} $$

where the first term $E\{\Pi (t,T)|\mathfrak {F}_{t}\}$ is the price under the assumption that both the investor and counterparty are default-free, and the second and third terms are the replacement costs. $\mathfrak {F}_{t}$ contains the full information before time *t*, LGD=(1−RecoverRate) is the loss given default, NPV(*t*) is net present value of the residual payoff for the investor until maturity from time *t*, *P*(*t*_1_,*t*_2_) is the price at *t*_1_ of a zero coupon bond matured at time *t*_2_, i.e. the discount rate from time *t*_1_ to *t*_2_.

In this case, CVA is $E\{\Pi (t,T)|\mathfrak {F}_{t}\}-E\left \{\Pi ^{D}(t,T)|\mathfrak {F}_{t}\right \}$.

If only counterparty risk is considered from the viewpoint of the bank (investor), the new price due to counterparty default is:

1 $$\begin{array}{*{20}l} E\left\{\Pi^{D}(t,T)|\mathfrak{F}_{t}\right\}&=E\left\{\Pi(t,T)|\mathfrak{F}_{t}\right\}\\ &\quad-E\left\{\text{LGD}_{C}\cdot I(\tau_{C}< T)\cdot P(t,\tau_{C})\cdot[\text{NPV}(\tau_{C})]^{+}|\mathfrak{F}_{t}\right\}~. \end{array} $$

Again, CVA is $E\{\Pi (t,T)|\mathfrak {F}_{t}\}-E\{\Pi ^{D}(t,T)|\mathfrak {F}_{t}\}$. Notice that CVA is always non-negative when only the counterparty risk is taken in to account. But if the bilateral counterparty risk exists, CVA also can be negative. More importantly, the above formulas are correct only when the swap expires at the defaults, or the counterparty of the replacement contract is default-free.

CCIRS is a contract which can cover the loss due to the counterparty default in an interest rate swap. Suppose the bank enters an interest-rate swap with a counterparty so that the bank receives from the counterparty a fixed amount periodically in exchange for the LIBOR linked floating payment from the bank. If the counterparty defaults, the bank needs to enter another swap agreement. However, the fixed rate will likely be different from the original one since the interest rate environment and number of remaining payments have changed. Thus, the bank bears the risk of making higher payment due to the possibility of default of the counterparty. There is also the possibility that in case of a default, the new rate is lower, but this scenario is of no concern to the bank from a risk management point of view. The purchase of a CCIRS eliminates that risk, and the fair price of CCIRS should be the expectation of the possible loss at the time when CCIRS is issued. Therefore, the pricing problem of CCIRS is equivalent to that of a CVA problem for interest rate swap when only counterparty risk is considered as in formula (1), under the assumption that the replacement contract is default-free. When the counterparty of the replacement contract is not default-free, the pricing formulae (1) underestimates the risk.

## CCIRS with default-free replacement contracts

To price a CCIRS, we first describe how an interest rate swap works and the relationship between the fixed and floating legs of the swap. A swap is a derivative contract in finance in which two counterparties enter an agreement to exchange certain benefits of one party’s financial instrument to another. The benefits in question depend on the type of financial instruments involved. Specifically, if the two counterparties agree to exchange one stream of interest rate payments against another stream of payments, the derivative is an interest rate swap. If the two counterparties sign an interest rate swap contract, then one counterparty agrees to make fixed payments at specified times. Normally the payment is the product of the notional value, the time interval between payments and the agreed fixed rate, i.e. *N* *o* *l*×*Δ* *t*×*R*_fixed_. In return, it will receive a stream of payments based on the floating rate. Similarly, the payment is normally the product of notional value *Nol*, the time between payments *Δ* *t* and the current floating rate *R*_floating_(*t*) (take in-arrears swap for example), which is usually an indexed reference rate (such as LIBOR) with a fixed spread *S*_*p*_ (can be 0). i.e. *N* *o* *l*×*Δ* *t*×(*R*_floating_(*t*)+*S*_*p*_). For example, a company signs an interest rate swap contract with a bank. The swap requires the company to pay a fixed rate at 5% in each payment time and the company receives a payment at the LIBOR rate in return. The notional value is $1,000,000. The maturity of the swap is five years and payment is made semi-annually. Every half a year, the company pays 1,000,000×0.5×5*%*=*$*25,000 and receives 1,000,000×0.5×*L* *I* *B* *O* *R*(*t*), where *t* is the time when payment is made.

### CCIRS pricing

In an interest rate swap, one party is required to make payments during each *Δ* *t* period, from *t*_1_ to *t*_*n*_. Let *t* be the current time, the (random) default time for the counterparty is *τ*, the next payment time is *t*_*k*_, the last payment time is *t*_*n*_ and *T* is the expiry time for the swap. *K* is the initial fixed rate on the fixed swap leg. If the default does not occur, the present value of the remaining payments at time *τ* is *N* *o* *l*·*A*_*τ*_(*τ*,*T*)*K*, where $A_{\tau }(\tau,T)=\Delta t_{i} \sum ^{n}_{i=k}P(\tau,t_{i})$ is the remaining annuity after time *τ* and *P*(*τ*,*t*_*i*_) is the *t*_*i*_-maturity zero coupon bond price at time *τ*. When the counterparty defaults at time *τ*, the payment of the replacement contract is *N* *o* *l*·*Δ* *t* *R*_*τ*_(*τ*,*T*), where *R*_*τ*_(*τ*,*T*) is the new fixed swap-rate calculated at time *τ*. The present value of the remaining payments (assuming no additional defaults) at time *τ* is *N* *o* *l*·*A*_*τ*_(*τ*,*T*)*R*_*τ*_(*τ*,*T*). Normally a fraction of the present value *Rec* can be recovered at default. Therefore, only the portion 1−*R* *e* *c* needs to be covered by CCIRS.

When the counterparty of the replacement contract is default-free, we can now write down the cost of replacing the swap. For the counterparty paying the fixed rate, the possible loss when *τ*<*T* is

$$\begin{array}{*{20}l} v(\tau)&=(1-Rec)\left(Nol\cdot A_{\tau}(\tau,T) R_{\tau}(\tau,T)- Nol\cdot A_{\tau}(\tau,T) K\right)^{+}\\ &=Nol\cdot (1-Rec) A_{\tau}(\tau,T) (R_{\tau}(\tau,T)-K)^{+}; \end{array} $$

while for the counterparty receiving the fixed rate, the possible loss is

$$\begin{array}{*{20}l} v(\tau)&=(1-Rec)\left(Nol\cdot A_{\tau}(\tau,T) K- Nol\cdot A_{\tau}(\tau,T) R_{\tau}(\tau,T)\right)^{+}\\ &=Nol\cdot (1-Rec) A_{\tau}(\tau,T) (K-R_{\tau}(\tau,T))^{+}. \end{array} $$

The derivative price at time *t* is simply the discounted expected value of *v*(*τ*) at time *t* under the risk-neutral measure, i.e., $v(t)=E\left [I_{\tau <T}\text {exp}\left (-\int ^{\tau }_{t}r(s)ds\right)v(\tau)|\mathfrak {F}_{t}\right ]$. In our case, we only considered the price of CCIRS when the investor is paying the fixed rate. The price at default time *τ* is

2 $$ v(\tau)=Nol\cdot (1-Rec) A_{\tau}(\tau,T) (R_{\tau}(\tau,T)-K)^{+}.  $$

The derivative price at time *t* is

3 $$ v(t)=Nol\cdot(1-Rec)\cdot E\left[\left.I_{\tau<T}\text{exp}\left(-\int^{\tau}_{t}r(s)ds\right) A_{\tau}(\tau,T) \left(R_{\tau}(\tau,T)-K\right)^{+}\right|\mathfrak{F}_{t}\right].  $$

Since both *Nol* and *Rec* are constants, we only need to compute the scaled price

4 $$  E\left[I_{\tau<T}\text{exp}\left(-\int^{\tau}_{t}r(s)ds\right) A_{\tau}(\tau,T) \left(R_{\tau}(\tau,T)-K\right)^{+}\Bigg|\mathfrak{F}_{t}\right].  $$

The final price can be obtained by multiplying *N* *o* *l*×(1−*R* *e* *c*).

### Model selection

Since the default time *τ* involves a hazard rate process, a proper model of this process needs to be chosen. On top of that, we need to choose a proper model for the interest rate as well. In our research, we assume the hazard rate process is the same for all counterparties with the same credit rating. Then we assume that both the interest rate and hazard rate follow the mean reverting Cox-Ingersoll-Ross (CIR) model (introduced in Cox et al. (1985) [17]), a widely used model in industry. CIR model is given by

5 $$ dr=a_{1}\left(b_{1}-r(t)\right)dt+\sigma_{1}\sqrt{r(t)}dB^{1}_{t}  $$

6 $$ d\lambda=a_{2}\left(b_{2}-\lambda(t)\right)dt+\sigma_{2}\sqrt{\lambda(t)}dB^{2}_{t}  $$

where $B^{1}_{t}$ and $B^{2}_{t}$ are Brownian motions and they are correlated as $d\left [B^{1}_{t},B^{2}_{t}\right ]=\rho dt$, i.e., the hazard and interest rates are correlated with coefficient *ρ*. When $2a_{1}b_{1}>\sigma _{1}^{2}$ and $2a_{2}b_{2}>\sigma _{2}^{2}$, this model ensures the interest rate and hazard rate are always positive and will never touch zero.

### Bond pricer under stochastic short rate

Denoting the price of zero coupon bond at time *t*_1_ which matures at time *t*_2_ is *P*(*t*_1_,*t*_2_), we expect

$$P\left(t_{1},t_{2}\right)=E\left[\left.\exp\left(-\int^{t_{2}}_{t_{1}}r(s)ds\right)\right|\mathfrak{F}_{t_{1}}\right], $$

where *r*(*s*) is short rate at time *s* and it follows:

$$dr=a_{1}(b_{1}-r(t))dt+\sigma_{1}\sqrt{r(t)}dB^{1}_{t}. $$

Since $\exp \left (-\int ^{t_{1}}_{r}r(s)ds\right)P(t_{1},t_{2})$ is a martingale, *P*(*t*_1_,*t*_2_) satisfies the partial differential equation (PDE)

7 $$ \partial_{t}P+a_{1}(b_{1}-r)\partial_{r}P+\frac{1}{2}\sigma_{1}^{2}r\partial_{rr}P=rP  $$

with terminal condition *P*(*t*_2_,*t*_2_)=1. This PDE is solved analytically as

8 $$ P(t_{1},t_{2})=\Lambda(t_{1},t_{2})\exp\left(-B(t_{1},t_{2})r(t)\right),  $$

where

$$\begin{array}{*{20}l} \Lambda(t_{1},t_{2})&=\left\{\frac{2\gamma \exp\left[\frac{1}{2}\left(a_{1}+\gamma\right)\left(t_{2}-t_{1}\right)\right]} {\left(\gamma+a_{1}\right)\left\{\exp\left[\gamma(t_{2}-t_{1})\right]-1\right\}+2\gamma}\right\}^{\frac{2a_{1}b_{1}}{\sigma_{1}^{2}}}, \\ B(t_{1},t_{2})&=\frac{2\left\{\exp\left[\gamma(t_{2}-t_{1})\right]-1\right\}} {\left(\gamma+a_{1}\right)\left\{\exp\left[\gamma(t_{2}-t_{1})\right]-1\right\}+2\gamma} \end{array} $$

with $\gamma =\sqrt {a_{1}^{2}+2\sigma _{1}^{2}}$.

### Partial differential equations

In this subsection, we derive the partial differential equations that are needed for pricing a CCIRS.

#### **Lemma 3.1**

Assuming *τ* is the first jump time of a Poisson process with the intensity process *λ*(*t*). Define *V*(*t*) as:

$$\begin{array}{*{20}l} V(t)&=E\left.\left[\text{exp}\left(-\int^{\tau}_{t}r(s)ds\right)f(\tau)\right|\mathfrak{F}_{t}\right] \end{array} $$

where *f* is a real function, $\mathfrak {F}_{t}=\mathfrak {G}_{t}\cup \sigma (I_{t>\tau }, 0\leq t\leq T)$. All processes relevant in determining values of the spot rate and the hazard rate of default are adapted to $\mathfrak {G}$. Use *E*_*t*_[∙] to represent $E[\bullet |\mathfrak {G}_{t}]$ for short.

Then we have:

$$\begin{array}{*{20}l} V(t) &=I_{t<\tau}E_{t}\left[\int_{t}^{T}f(s)\lambda(s)\text{exp}\left(-\int^{s}_{t}(r(u) +\lambda(u))du\right)ds\right] \end{array} $$

#### *Proof*

See [18], Prop 3.1. □

The value of CCIRS at time *t* can be represented as

$$v(t)=E\left.\left[I_{\tau< T}\text{exp}\left(-\int^{\tau}_{t}r(s)ds\right) A_{\tau}(\tau,T)(R_{\tau}(\tau,T)-K)^{+}\right|\mathfrak{F}_{t}\right]. $$

Actually the PDE of *v*(*t*) is the direct result of Feynman-Kac theorem. We decided to elaborate the proof here to make our paper more complete.

#### **Theorem 3.2**

Define *F*(*t*) as:

$${}F(t)=v(t)\text{exp}\left(-\int^{t}_{0}(r(u)+\lambda(u))du\right)+\int_{0}^{t}f(s)\lambda(s) \text{exp}\left(-\int^{s}_{0}(r(u)+\lambda(u))du\right)ds, $$

where

$$f(t) = I_{t< T}A_{t}(t,T)\left(R_{t}(t,T)-K\right)^{+}. $$

Here *R*_*t*_(*t*,*T*) is the fixed rate for an interest rate swap, signed at time t, maturing at time T.

Then *F*(*t*) is a martingale.

#### *Proof*

Use Lemma 3.1, we have

9 $$\begin{array}{*{20}l} v(t)=E_{t}\left[\int_{t}^{T}f(s)\lambda(s)\text{exp}\left(-\int^{s}_{t}(r(u)+\lambda(u))du\right)ds\right], \end{array} $$

which gives

$$\begin{array}{*{20}l} {}F(t)&=v(t)\text{exp}\left(-\int^{t}_{0}(r(u)+\lambda(u))du\right)+\int_{0}^{t}f(s)\lambda(s) \text{exp}\left(-\int^{s}_{0}(r(u)+\lambda(u))du\right)ds\\ &=E_{t}\left[\int_{0}^{T}f(s)\lambda(s) \text{exp}\left(-\int^{s}_{0}(r(u)+\lambda(u))du\right)ds\right]. \end{array} $$

Notice the last expression above is a martingale since the expectation does not contain *t*. We denote the function inside the expectation as *H*. This means *F*(*t*)=*E*_*t*_(*H*). Given the definition of a martingale, we only need to show

1. *E*_*t*_(|*H*|)<*∞*;
2. *E*_*t*_[*F*(*s*)]=*F*(*t*),(*s*>*t*).

The first inequality is true evidently. Since

$$\begin{array}{*{20}l} E_{t}\left[F(s)\right]&=E_{t}\left[E_{s}(H)\right] =E_{t}\left(H\right)=F(t), \end{array} $$

*F*(*t*) is a martingale. □

#### **Theorem 3.3**

The PDE satisfied by *v*(*t*,*r*,*λ*) is

10 $$ \left(\partial_{t}+\mathcal{L}\right)v+\lambda(f-v)-rv=0  $$

with terminal condition *v*(*T*,*r*,*λ*)=0, where

$$\begin{array}{*{20}l} \mathcal{L}&=a_{1}(b_{1}-r)\partial_{r}+\frac{1}{2}\sigma^{2}_{1}r \partial_{rr}+a_{2}(b_{2}-\lambda)\partial_{\lambda}+\frac{1}{2}\sigma^{2}_{2}\lambda \partial_{\lambda\lambda}+\rho\sigma_{1}\sigma_{2}\sqrt{r\lambda}\partial_{r\lambda}, \\ f&=A_{t}(t,T)(R_{t}(t,T)-K)^{+} \end{array} $$

#### *Proof*

To simplify notation, we denote

$${}\hat{D}(t)=\text{exp}\!\left(-\!\int^{t}_{0}(r(u)+\lambda(u))du\right),\quad \!\!M(t)=\!\int_{0}^{t}f(s)\lambda(s) \text{exp}\left(-\int^{s}_{0}(r(u)+\lambda(u))du\right)ds $$

and $F(t)=v(t)\hat {D}(t)+M(t).$ Recall the models for *r* and *λ*

$$dr=a_{1}(b_{1}-r(t))dt+\sigma_{1}\sqrt{r(t)}dB^{1}_{t}, \quad d\lambda=a_{2}(b_{2}-\lambda(t))dt+\sigma_{2}\sqrt{\lambda(t)}dB^{2}_{t}. $$

Applying Ito’s lemma

$$d\hat{D}(t)=-\hat{D}(t)(r(t)+\lambda(t))dt,\quad dM(t)=f(t)\lambda(t)\hat{D}(t)dt, $$

and

$$\begin{array}{*{20}l} dv&=v_{t}dt+v_{r}dr+v_{\lambda} d\lambda+\frac{1}{2}v_{rr}drdr+v_{r\lambda}drd\lambda+\frac{1}{2}v_{\lambda\lambda}d\lambda d\lambda, \\ &=v_{t}dt+v_{r}\left(a_{1}(b_{1}-r)dt+\sigma_{1}\sqrt{r}dB^{1}_{t}\right)+v_{\lambda}\left(a_{2}(b_{2}-\lambda) dt+\sigma_{2}\sqrt{\lambda} dB^{2}_{t}\right)\\ &\quad+\frac{1}{2}v_{rr}\sigma_{1}^{2}rdt+\rho \sigma_{1}\sigma_{2}\sqrt{r\lambda} v_{r\lambda}dt+\frac{1}{2}v_{\lambda\lambda}\sigma_{2}^{2}dt\\ &=(\partial_{t}+\mathcal{L})vdt+v_{r}\sigma_{1}\sqrt{r}dB^{1}_{t}+v_{\lambda}\sigma_{2}\sqrt{\lambda} dB^{2}_{t}. \end{array} $$

This leads to

$$\begin{array}{*{20}l} dF(t)&=v(t)d\hat{D}(t)+\hat{D}(t)dv+dM(t)\\ &=-v(t)\hat{D}(t)(r(t)+\lambda(t))dt\\ &\quad+\hat{D}(t)\left(\left(\partial_{t}+\mathcal{L}\right)vdt+v_{r}\sigma_{1}\sqrt{r}{dB}_{t}^{1}+v_{\lambda} \sigma_{2}\sqrt{\lambda}{dB}_{t}^{2}\right)+f(t)\lambda(t)\hat{D}(t)dt\\ &=\hat{D}(t)\left[\left(\partial_{t}+\mathcal{L}\right)v-v\left(r+\lambda\right)+f\lambda\right] dt+\hat{D}(t)v_{r}\sigma_{1}\sqrt{r}{dB}_{t}^{1}+\hat{D}(t)v_{\lambda}\sigma_{2}\sqrt{\lambda} {dB}_{t}^{2}. \end{array} $$

From Theorem 3.2, we know *F*(*t*) is a martingale. Therefore the coefficient of the *dt* term in *d* *F*(*t*) must vanish, which gives

$$\left(\partial_{t}+\mathcal{L}\right)v-v(r+\lambda)+f\lambda=0, $$

which can be rearranged to

$$\left(\partial_{t}+\mathcal{L}\right)v+\lambda(f-v)-rv=0. $$

If the counterparty defaults at or after the maturity *T*, there is no need to replace the original swap. In this case, the price of CCIRS is zero. This gives us the terminal condition as

11 $$ v(T,\lambda, r)=0.  $$

□

In order to solve *v*(*t*,*r*,*λ*), the remaining task is to find the associated boundary conditions. With the terminal condition we know this PDE can be solved backward as long as we find the proper boundary condition, which we can achieve by looking at the characteristic function of *r* and *λ*.

For the lower boundary of *r*, ie., *r*=0, we have $\frac {dr}{dt}=a_{1} b_{1}>0$. For the upper boundary of *r*, ie., *r*=*∞*, we have $\frac {dr}{dt}=a_{1}(b_{1}-r)+\sigma _{1}\sqrt {r}{dB}_{t}^{1}/dt$. The term $\sigma _{1}\sqrt {r}{dB}_{t}^{1}/dt$ can be ignored because its order is $\sqrt {r}$ and *a*_1_(*b*_1_−*r*) has the order *r*. So $\frac {dr}{dt}\approx a_{1}(b_{1}-r)<0$. These two characteristic function values on the boundary line ensure that when time decreases from the terminal line (*t*=*T*), the value of *r*(*t*) goes towards the boundary lines when *r*(*t*) is close to them. Similarly, the behavior of the value respect to *λ*(*t*) is the same near its boundary lines *λ*=0 and *λ*=*∞*. By looking at these characteristic function values of *r* and *λ* close to the boundary lines, we know it will be a good approximation to replace the derivative on the boundary lines with the derivative of the inside point beside the boundary lines. Then with this boundary condition, the PDE (10) can be solved numerically.

## CCIRS with defaultable replacement contract

In the previous section, when the counterparty defaults and a new replacement swap contract is signed, it was assumed that the counterparty of the new contract is default-free. Therefore, the CCIRS price obtained in the previous section is only an approximation, which may underestimate the real price. This is justified for a counterparty with a high credit rating when the time to maturity is short. In practice, however, the time to maturity of these contracts is relatively long (e.g., 10 years). Therefore, it will be of practical interest to investigate the effect of the default-free assumption, which is the focus of this section.

We assume that the replacement contract could also default but its replacement is default-free. In the rest of the paper, this is called “two-default” problem, which is a more accurate approximation of the time cost than the default-free replacement contract model, or the “one-default" problem discussed previously. In addition we make two more assumptions: first, the hazard rate process is for a certain credit rating; second, the replacement has to have the same credit ratings as the original contract when it defaults. Under those two assumptions, the “second" default problem is actually conditional on the occurrence of the first default in the same Poisson process. Let *τ*_1_ and *τ*_2_ (*τ*_2_>*τ*_1_) be the default times of the original and replacement counterparties, respectively. They are the first and the second jumps time of the Cox process with hazard rate *λ* given by (6). Recall that the price of CCIRS with a default-free replacement contractor is given by (4) as

$$V(t)=Nol\cdot(1-Rec) E\left.\left[I_{\tau<T}\exp\left(-\int^{\tau_{1}}_{t}r(s)ds\right) A_{\tau_{1}}\left({\tau_{1}},T\right) \left(R_{\tau_{1}}\left({\tau_{1}},T\right)-K\right)^{+}\right|\mathfrak{F}_{t}\right]. $$

We can rewrite this equation as:

12 $$ V(t)=Nol\cdot(1-Rec) E\left.\left[D\left(t,\tau_{1}\right)f\left(\tau_{1}\right)\right|\mathfrak{F}_{t}\right]~,  $$

where

$$f(\tau_{1})=\left\{ \begin{array}{ll} A_{\tau_{1}}\left({\tau_{1}},T\right) \left(R_{\tau_{1}}\left({\tau_{1}},T\right)-K\right)^{+}, & \tau_{1}<T~; \\ 0, & \tau_{1}\geq T, \end{array} \right. $$

and

$$D\left(t,\tau_{1}\right)=\text{exp}\left[-\int^{\tau_{1}}_{t}r(s)ds\right]~. $$

Again, *N* *o* *l*·(1−*R* *e* *c*) is a constant which we will drop in the following discussion knowing that the final price can be obtained by multiplying our numerical solution with this constant. When the counterparty of the replacement contractor is allowed to default, there are three scenarios.

(i). Only one default occurs before maturity. Based on Equation (2), the loss at the first default *τ*_1_ is
  $$v(\tau_{1})= A_{\tau_{1}}(\tau_{1},T) \left(R_{\tau_{1}}(\tau_{1},T)-K\right)^{+} $$
(ii). Both defaults occur before maturity. The fixed rate for a new swap at *τ*_2_ is $R_{\tau _{2}}({\tau _{2}},T)$. The fixed rate payment of the replacement swap between *τ*_1_ and *τ*_2_ is $\Delta {tR}_{\tau _{1}}({\tau _{1},T})$ and the fixed rate payment of the second replacement swap between *τ*_2_ and *T* is $\Delta {tR}_{\tau _{2}}({\tau _{2}},T)$. The discounted value of all the payments between *τ*_1_ and *τ*_2_ is $A_{\tau _{1}}({\tau _{1}},\tau _{2}) R_{\tau _{1}}({\tau _{1},T})$. The discounted value of all the payments between *τ*_2_ and *T* is $\phantom {\dot {i}\!}D(\tau _{1},\tau _{2}){NA}_{\tau _{2}}({\tau _{2}},T) R_{\tau _{2}}({\tau _{2}},T)$. The value of CCIRS at time *τ*_1_ is the sum
  $$v(\tau_{1})= A_{\tau_{1}}\left({\tau_{1}},\tau_{2}\right) \left(R_{\tau_{1}}\left({\tau_{1}},T\right)-K\right)^{+} +A_{\tau_{2}}\left({\tau_{2}},T\right) \left(R_{\tau_{2}}\left({\tau_{2}},T\right)-K\right)^{+}D\left(\tau_{1},\tau_{2}\right). $$
(iii). The first default happens after maturity. There is no cost and the value of CCIRS is zero.

Considering all cases above, the CCIRS price is given by

$$W(t)=E\left.\left[D\left(t,\tau_{1}\right)f\left(\tau_{1},\tau_{2}\right)\right|\mathfrak{F}_{t}\right] $$

where

$$f\left(\tau_{1},\tau_{2}\right)=\left\{ \begin{array}{ll} 0 & \tau_{2}>\tau_{1}>T;\\ A_{\tau_{1}}\left({\tau_{1}},T\right) \left(R_{\tau_{1}}\left({\tau_{1}},T\right)-K\right)^{+} & \tau_{2}>T>\tau_{1};\\ A_{\tau_{1}}\left({\tau_{1}},\tau_{2}\right) \left(R_{\tau_{1}}\left({\tau_{1}},T\right)-K\right)^{+} & \\ \qquad\qquad+ A_{\tau_{2}}\left({\tau_{2}},T\right) \left(R_{\tau_{2}}\left({\tau_{2}},T\right)-K\right)^{+}D\left(\tau_{1},\tau_{2}\right) &T>\tau_{2}>\tau_{1}. \end{array} \right. $$

To simplify notation, let

13 $$ \widetilde{A}(t_{1},t_{2})=\left\{ \begin{array}{ll} A_{t_{1}}(t_{1},t_{2}) & \hbox{\(T>t_{2}>t_{1}\);} \\ A_{t_{1}}(t_{1},T) & \hbox{\(t_{2}>T>t_{1}\);} \\ 0 & \hbox{otherwise.} \end{array} \right.  $$

We rewrite *f*(*τ*_1_,*τ*_2_) as

$$\widetilde{A}\left(\tau_{1},\tau_{2}\right)\left(R_{\tau_{1}}({\tau_{1}},T)-K\right)^{+} +\widetilde{A}(\tau_{2},T)\left(R_{\tau_{2}}({\tau_{2}},T)-K\right)^{+}D(\tau_{1},\tau_{2}) $$

and *W*(*t*) can be written as

14 $$\begin{array}{*{20}l} W(t)&=E\Big\{D(t,\tau_{1})\Big[\widetilde{A}(\tau_{1},\tau_{2})(R_{\tau_{1}}({\tau_{1}},T)-K)^{+}\\ &\quad+\widetilde{A}(\tau_{2},T)(R_{\tau_{2}}({\tau_{2}},T)-K)^{+}D(\tau_{1},\tau_{2})\Big]\Big|\mathfrak{F}_{t}\Big\}. \end{array} $$

To derive the PDE for *W*(*t*), we need the following theorems and corollaries.

### **Corollary 4.1**

For any *T*>0 and *τ*>*t*, let *Z*_*t*_ be a $\mathfrak {G}_{t}$-adapted stochastic process and *Z*_*t*_≢0 when *t*≥*T*, then:

$$E\left.\left[D(t,\tau)Z_{\tau}\right.|\mathfrak{F}_{t}\right]=E_{t}\left[\int^{+\infty}_{t}Z_{s}\lambda_{s}\hat{D}(t,s)ds\right]. $$

### *Proof*

Denote *t*_*i*_=*t*+*i* *Δ* *t*, *i*=0,1,.... We have $Z^{(i)}_{s}=Z_{s}I_{t_{i}\leq s<t_{i+1}}$ and $Z_{s}=\sum _{i=0}^{\infty } Z^{(i)}_{s}$. It follows that

$$\begin{array}{*{20}l} E\left[\left.D(t,\tau)Z_{\tau}\right|\mathfrak{F}_{t}\right]&=E\left[D(t,\tau)\sum_{i=0}^{\infty} Z^{(i)}_{\tau}\bigg|\mathfrak{F}_{t}\right]\\ (\text{Since}~ D(t,\tau)Z^{(i)}_{\tau}\geq 0,& \mathrm{~by~Tonelli's~Theorem,~we ~have})\\ &=\sum_{i=0}^{\infty} E\left[D(t,\tau)Z^{(i)}_{\tau}\bigg|\mathfrak{F}_{t}\right]. \end{array} $$

Since each $Z^{(i)}_{\tau }=0$ when *τ*≥*t*_*i*+1_, Lemma 3.1 applies and

15 $$\begin{array}{*{20}l} \sum_{i=0}^{\infty} E\left[D(t,\tau)Z^{(i)}_{\tau}\bigg|\mathfrak{F}_{t}\right]&=\sum_{i=0}^{\infty} I_{\tau\geq  t}E_{t}\left[\int^{t_{i+1}}_{t}Z_{s}^{(i)}\lambda_{s}\hat{D}(t,s)ds\right]\\ &=\sum_{i=0}^{\infty} I_{\tau\geq t}E_{t}\left[\int^{t_{i+1}}_{t}Z_{s}I(t_{i}\leq s<t_{i+1})\lambda_{s}\hat{D}(t,s)ds\right]\\ &=I_{\tau\geq t}\sum_{i=0}^{\infty} E_{t}\left[\int^{t_{i+1}}_{t_{i}}Z_{s}\lambda_{s}\hat{D}(t,s)ds\right]\\ & (\text{Since}~ \int^{t_{i+1}}_{t_{i}}Z_{s}\lambda_{s}\hat{D}(t,s)ds\geq 0, \mathrm{~by~Tonelli's~ Theorem})\\ &=I_{\tau\geq t}E_{t}\left[\sum_{i=0}^{\infty} \int^{t_{i+1}}_{t_{i}}Z_{s}\lambda_{s}\hat{D}(t,s)ds\right]\\ &=I_{\tau\geq t}E_{t}\left[\int^{+\infty}_{t}Z_{s}\lambda_{s}\hat{D}(t,s)ds\right]. \end{array} $$

This proves the corollary. □

### **Corollary 4.2**

From Corollary 4.1, let *r*_*t*_≡0, for any *τ*>*t* we have

$$E\left[\left.Z_{\tau}\right|\mathfrak{F}_{t}\right]=E_{t}\left[\int^{+\infty}_{t}Z_{s}\lambda_{s} exp\left[-\int_{t}^{s}\lambda(k)dk\right]ds\right]. $$

### **Corollary 4.3**

From Corollary 4.1, let *r*_*t*_≡0 and *Z*(*t*)≡1, for any *τ*>*t* we have

$$1=E_{t}\left[\int^{+\infty}_{t}\lambda_{s}\text{exp}\left(-\int_{t}^{s}\lambda_{u}du\right) ds\right]. $$

### **Theorem 3.4**

(This is a stronger result than Corollary 4.3.) It is a reasonable assumption that *λ*(*t*) is always positive; then we have:

$$\int_{t}^{\infty}\lambda_{s}\text{exp}\left(-\int_{t}^{s}\lambda_{u}du\right)ds=1. $$

### *Proof*

From the proof of Proposition 3.1 in [18], the density of the default time for *s*>*t* is given by

$$\frac{\partial}{\partial s}\mathrm{P}\left(\tau\leq s|\tau>t,\mathfrak{G}_{T}\right)=\lambda_{s}\text{exp}\left(-\int_{t}^{s}\lambda_{u}du\right). $$

We know the integration of density function is 1, which proves Theorem 3.4. □

### **Theorem 3.5**

$$A_{u}(u,s)=A_{u}(u,T) -E[D(u,s)A_{s}(s,T)|\mathfrak{F}_{u}]. $$

### *Proof*

First, we have

$$\begin{array}{*{20}l} A_{u}(u,T)-A_{u}(u,s)=\Delta t\sum^{n}_{i=k_{u}}P(u,t_{i})-\Delta t\sum^{j_{s}}_{i=k_{u}}P(u,t_{i}), =\Delta t\sum^{n}_{i=j_{s}+1}P(u,t_{i}). \end{array} $$

where *k*_*u*_ is the next payment time after time *u*, and *j*_*s*_ is the closest payment time which is before or equal to *s*.

Since

$$P(u,t_{i})=E[D(u,s)D(s,t_{i})|\mathfrak{F}_{u}], $$

we have

$$\begin{array}{*{20}l} \Delta t\sum^{n}_{i=j_{s}+1}P(u,t_{i})&=E\left.\left[\Delta t\sum^{n}_{i=j_{s}+1}D(u,s)D(s,t_{i})\right|\mathfrak{F}_{u}\right]\\ &=E\left.\left[D(u,s)\Delta {tE}_{s}\left[\sum^{n}_{i=j_{s}+1}D(s,t_{i})\right]\right|\mathfrak{F}_{u}\right]\\ &=E\left.\left[D(u,s)\Delta t\sum^{n}_{i=j_{s}+1}P(s,t_{i})\right|\mathfrak{F}_{u}\right] \\ &=E\left.\left[D(u,s)A_{s}(s,T)\right|\mathfrak{F}_{u}\right]. \end{array} $$

□

### **Corollary 4.4**

$$\widetilde{A}(u,s)=\widetilde{A}(u,T) -E[D(u,s)\widetilde{A}(s,T)|\mathfrak{F}_{u}]\qquad (s>u). $$

### *Proof*

For *s*>*T*, the left-hand-side equals to $\widetilde {A}(u,T)$, and the right-hand-side equals to $\widetilde {A}(u,T)-0$. Therefore the Corollary is true. For *u*>*T*, both sides of the equation equal to zero. Finally, for *s*<*T*, the left-hand-side equals to *A*(*u*,*s*) and the right-hand-side equals to $A_{u}(u,T) -E[D(u,s)A_{s}(s,T)|\mathfrak {F}_{u}]$. Applying Theorem 3.5 proves the Corollary. □

With these preparations, we are now in the position to derive the PDE for *W*(*t*). We note that

$$\begin{array}{*{20}l} W(t)&=E\Big\{D(t,\tau_{1})\Big[\widetilde{A}\left(\tau_{1},\tau_{2}\right)\left(R_{\tau_{1}}\left({\tau_{1}},T\right)-K\right)^{+}\\ &\quad+\widetilde{A}(\tau_{2},T)\left(R_{\tau_{2}}({\tau_{2}},T)-K\right)^{+}D(\tau_{1},\tau_{2})\Big]\Big|\mathfrak{F}_{t}\Big\}\\ &=E\Big\{D(t,\tau_{1})E\Big[\widetilde{A}(\tau_{1},\tau_{2})\left(R_{\tau_{1}}({\tau_{1}},T)-K\right)^{+}\\ &\quad+\widetilde{A}(\tau_{2},T)\left(R_{\tau_{2}}({\tau_{2}},T)-K\right)^{+} D(\tau_{1},\tau_{2})\big|\mathfrak{F}_{\tau_{1}}\Big]\Big|\mathfrak{F}_{t}\Big\}\\ &=E\Big\{D(t,\tau_{1})E\Big[\widetilde{A}\left(\tau_{1},\tau_{2}\right) \left(R_{\tau_{1}}({\tau_{1}},T)-K\right)^{+}\big|\mathfrak{F}_{\tau_{1}}\Big]\Big|\mathfrak{F}_{t}\Big\}\\ &\quad+E\Big\{D(t,\tau_{1})E\Big[\widetilde{A}(\tau_{2},T)(R_{\tau_{2}}({\tau_{2}},T)-K)^{+}D(\tau_{1},\tau_{2})\Big|\mathfrak{F}_{\tau_{1}}\Big]\Big|\mathfrak{F}_{t}\Big\}, \end{array} $$

which can be separated into two parts as

16 $$\begin{array}{*{20}l} W_{A}(t)=E\Big\{D(t,\tau_{1})E\Big[\widetilde{A}(\tau_{1},\tau_{2}) (R_{\tau_{1}}({\tau_{1}},T)-K)^{+}\big|\mathfrak{F}_{\tau_{1}}\Big]\Big|\mathfrak{F}_{t}\Big\}, \end{array} $$

and

17 $$\begin{array}{*{20}l} W_{B}(t)=E\Big\{D(t,\tau_{1})E\Big[\widetilde{A}(\tau_{2},T)\left(R_{\tau_{2}}({\tau_{2}},T)-K\right)^{+} D(\tau_{1},\tau_{2})\big|\mathfrak{F}_{\tau_{1}}\Big]\Big|\mathfrak{F}_{t}\Big\}. \end{array} $$

### PDE for *W*_*A*_(*t*)

From the definition of $\widetilde {A}(t_{1},t_{2})$ in (13), when *τ*_2_>*T*, $\widetilde {A}(\tau _{1},\tau _{2})=A_{\tau _{1}}(\tau _{1},T)\not \equiv 0$. Using Corollary 4.2 yields

$$\begin{array}{*{20}l} E\big[\widetilde{A}(\tau_{1},\tau_{2})\big|\mathfrak{F}_{\tau_{1}}\big]&=I_{\tau_{2}>\tau_{1}}E_{\tau_{1}}\left[\int^{\infty}_{\tau_{1}}\widetilde{A}(\tau_{1},s)\lambda(s)exp\left[-\int_{\tau_{1}}^{s}\lambda(k)dk\right]ds\right]\\ &=E_{\tau_{1}}\left[\int^{\infty}_{\tau_{1}}\widetilde{A}(\tau_{1},s)\lambda(s)exp\left[-\int_{\tau_{1}}^{s}\lambda(k)dk\right]ds\right] \end{array} $$

since *τ*_2_>*τ*_1_. Denote

$$l(u)=E_{u}\left[\int^{\infty}_{u}\widetilde{A}(u,s)\lambda(s)\exp\left(-\int_{u}^{s}\lambda(k)dk\right)ds\right], $$

and note that *l*(*u*)=0 when *u*≥*T*, due to $\widetilde {A}(T,s)\equiv 0$ by the definition of $\widetilde {A}(t_{1},t_{2}).$ With this new notation, we have

$$\begin{array}{*{20}l} W_{A}(t)&=E\Big\{D(t,\tau_{1})(R_{\tau_{1}}({\tau_{1}},T)-K)^{+}E\big[\widetilde{A}(\tau_{1},\tau_{2})|\mathfrak{F}_{\tau_{1}}\big]\Big|\mathfrak{F}_{t}\Big\}\\ &=E\Big[D(t,\tau_{1})(R_{\tau_{1}}({\tau_{1}},T)-K)^{+}l(\tau_{1})\Big|\mathfrak{F}_{t}\Big] \\ &=E_{t}\left[\int_{t}^{T}D(t,s)\lambda(s)(R_{s}({s},T)-K)^{+}l(s)ds\right]. \end{array} $$

Since $D(0,t)W_{A}(t)+\int _{0}^{t}D(t,s)\lambda (s)(R_{s}({s},T)-K)^{+}l(s)ds$ is a martingale. we obtain the PDE for *W*_*A*_(*t*) as

18 $$ (\partial_{t}+\mathcal{L})W_{A}+\lambda(f-W_{A})-{rW}_{A}=0  $$

with *W*_*A*_(*T*,*r*,*λ*)=0, where *f*=*l*(*t*)(*R*_*t*_(*t*,*T*)−*K*)^+^ and

$$\mathcal{L}=a_{1}(b_{1}-r)\partial_{r}+\frac{1}{2}\sigma^{2}_{1}r \partial_{rr}+a_{2}(b_{2}-\lambda)\partial_{\lambda}+\frac{1}{2}\sigma^{2}_{2}\lambda \partial_{\lambda\lambda}+\rho\sigma_{1}\sigma_{2}\sqrt{r\lambda}\partial_{r\lambda}. $$

By Corollary 4.4, we have

$$\begin{array}{*{20}l} l(u)&=E_{u}\left[\int^{\infty}_{u}(\widetilde{A}(u,T) -D(u,s)\widetilde{A}(s,T))\lambda(s)\text{exp}\left(-\int_{u}^{s}\lambda(k)dk\right)ds\right]\\ &=E_{u}\left[\int^{\infty}_{u}\widetilde{A}(u,T)\lambda(s)\text{exp}\left(-\int_{u}^{s}\lambda(k)dk\right)ds\right]\\ &\qquad-E_{u}\left[\int^{\infty}_{u}\widetilde{A}(s,T)\lambda(s)D(u,s)\text{exp}\left(-\int_{u}^{s}\lambda(k)dk\right)ds\right]\\ &=\widetilde{A}(u,T)E_{u}\left[\int^{\infty}_{u}\lambda(s)\text{exp}\left(-\int_{u}^{s}\lambda(k)dk\right)ds\right]\\ &\qquad-E_{u}\left[\int^{\infty}_{u}\widetilde{A}(s,T)\lambda(s)\hat{D}(u,s)ds\right]. \end{array} $$

By Theorem 3.4, we have $E_{u}\left [\int ^{\infty }_{u}\lambda (s)\text {exp}\left (-\int _{u}^{s}\lambda (k)dk\right)ds\right ]=1$, then

$$l(u)=\widetilde{A}(u,T)-E_{u}\left[\int^{\infty}_{u}\widetilde{A}(s,T)\lambda(s)\hat{D}(u,s)ds\right]. $$

Denote

$$h(u)=E_{u}\left[\int^{\infty}_{u}\widetilde{A}(s,T)\lambda(s)\hat{D}(u,s)ds\right]. $$

Since $\widetilde {A}(s,T)=0$ for *s*>*T*, and $\widetilde {A}(s,T)=A_{s}(s,T)$ for *s*<*T*, we have

$$h(u)=E_{u}\left[\int^{\infty}_{u}A_{s}(s,T)\lambda(s)\hat{D}(u,s)ds\right]. $$

It can be verified that

$$\hat{D}(0,u)h(u)+\int_{0}^{u}\hat{D}(0,s)\lambda(s)A_{s}(s,T)ds $$

is a martingale, which yields the PDE for *h*(*u*) as

19 $$ (\partial_{t}+\mathcal{L})h+\lambda(f-h)-rh=0  $$

with *h*(*T*,*r*,*λ*)=0, where *f*=*A*_*t*_(*t*,*T*) and

$$\mathcal{L}=a_{1}(b_{1}-r)\partial_{r}+\frac{1}{2}\sigma^{2}_{1}r \partial_{rr}+a_{2}(b_{2}-\lambda)\partial_{\lambda}+\frac{1}{2}\sigma^{2}_{2}\lambda \partial_{\lambda\lambda}+\rho\sigma_{1}\sigma_{2}\sqrt{r\lambda}\partial_{r\lambda}. $$

After we obtain *h*(*t*), we can find *l*(*t*) using $l(t)=\widetilde {A}(t,T)-h(t)$ and solve the PDE for *W*_*A*_(*t*).

### PDE for of *W*_*B*_(*t*)

From (17), we have

$$\begin{array}{*{20}l} W_{B}(t)&=E\left[D(t,\tau_{2}) \widetilde{A}(\tau_{2},T) (R_{\tau_{2}}({\tau_{2}},T)-K)^{+}\Big|\mathfrak{F}_{t}\right]\\ &=E\left[D(t,\tau_{1})E\left[\widetilde{A}(\tau_{2},T) (R_{\tau_{2}}({\tau_{2}},T)-K)^{+}P(\tau_{1},\tau_{2})\Big|\mathfrak{F}_{\tau_{1}}\right]\Big|\mathfrak{F}_{t}\right]\\ &=E\left[D(t,\tau_{1})E_{\tau_{1}}\left[\int^{T}_{\tau_{1}}\widetilde{A}(s,T) (R_{s}({s},T)-K)^{+}\lambda(s)D(\tau_{1},s)ds\right]\Big|\mathfrak{F}_{t}\right]. \end{array} $$

Here we have used Lemma 3.1. Let $p(u)=E_{u}\left [\int ^{T}_{u}\widetilde {A}(s,T) (R_{s}(s,T)-K)^{+}\lambda (s)D(u,s)ds\right ]$. When *s*≤*T*, since $\widetilde {A}(s,T)=A_{s}(s,T)$ by definition, we can rewrite *p*(*u*) as

$$p(u)=E_{u}\left[\int^{T}_{u}A_{s}(s,T) (R_{s}(s,T)-K)^{+}\lambda(s)D(u,s)ds\right].$$

Thus, we obtain

$$W_{B}(t)=E\left[D(t,\tau_{1})p(\tau_{1})\big|\mathfrak{F}_{t}\right] $$

And by Lemma 3.1, we can write *W*_*B*_(*t*) as

$$W_{B}(t)=E_{t}\left[\int_{t}^{T}D(t,s)\lambda(s)p(s)ds\right] $$

It can be verified that $D(0,t)g(t)+\int _{0}^{t}D(t,s)\lambda (s)p(s)ds$ is a martingale, from which we obtain the PDE for *W*_*B*_(*t*) as

20 $$ (\partial_{t}+\mathcal{L})W_{B}+\lambda(p-W_{B})-{rW}_{B}=0  $$

with *W*_*B*_(*T*,*r*,*λ*)=0, where

$$\mathcal{L}=a_{1}(b_{1}-r)\partial_{r}+\frac{1}{2}\sigma^{2}_{1}r \partial_{rr}+a_{2}(b_{2}-\lambda)\partial_{\lambda}+\frac{1}{2}\sigma^{2}_{2}\lambda \partial_{\lambda\lambda}+\rho\sigma_{1}\sigma_{2}\sqrt{r\lambda}\partial_{r\lambda}. $$

Since $p(u)=E_{u}\left [\int ^{T}_{u}A_{s}(s,T) (R_{s}(s,T)-K)^{+}\lambda (s)D(u,s)ds\right ]$ is defined similarly as the value in Eq. (9), we can derive the PDE for *p*(*u*) in a similar way, which is given by

21 $$ (\partial_{t}+\mathcal{L})p+\lambda(f-p)-rp=0  $$

with *p*(*T*,*r*,*λ*)=0, where

$$\mathcal{L}=a_{1}(b_{1}-r)\partial_{r}+\frac{1}{2}\sigma^{2}_{1}r \partial_{rr}+a_{2}(b_{2}-\lambda)\partial_{\lambda}+\frac{1}{2}\sigma^{2}_{2}\lambda \partial_{\lambda\lambda}+\rho\sigma_{1}\sigma_{2}\sqrt{r\lambda}\partial_{r\lambda}~, $$

$$f=A_{t}(t,T)(R_{t}(t,T)-K)^{+}. $$

### CCIRS price *W*(*t*)

We solve *W*_*A*_(*t*,*r*,*λ*) using two PDEs (18)-(19) and *W*_*B*_(*t*,*r*,*λ*) using (20)-(21) numerically with the ADI finite difference method. We can then obtain the final CCIRS price using *W*(*t*,*r*,*λ*)=*W*_*A*_(*t*,*r*,*λ*)+*W*_*B*_(*t*,*r*,*λ*).

## Numerical results

Before solving the partial differential equations, we will first estimate parameter values of the CIR model using historic data.

### Parameter values

For the interest rate, it is widely accepted that the risk-free rate curve is the best approximation of short rate. Before the 2008 financial crisis, LIBOR rate curve was often used when a risk-free curve is needed. The environment changed after the crisis and overnight indexed swap (OIS) rate is considered as a close approximation of the risk-free rate in many banks. However, the most appropriate estimation of the risk-free curve is not the focus of our paper and the data of OIS curve is not published, we use the LIBOR curve as the risk-free curve in this paper. Theoretically, if all LIBOR curves are risk-free, they should be identical if compounded to an annual rate. Here, it is reasonable to choose the 12 Month LIBOR curve as the approximation of the risk-free curve. The time frame for our chosen 5-year of data starts from May 1, 2009 to April 30, 2014.

Partial data of 12 Month LIBOR rate has been given in Table 1. We used the rate 0.5490% on April 30, 2014 as the initial rate. The mean is used as the long term average, i.e.,

$$b_{1}=mean(\text{data})=0.909\% $$

and

$$mean(\text{data})=\frac{\sum^{N}_{i=1}r_{i}}{N}, $$

where *N* is the number of data in five years and *r*_*i*_ is the 12 Month LIBOR in *i*th day.

**Table 1:** Twelve Month LIBOR Rate and CDS spread of different ratings (from May 1, 2009 to April 30, 2014)

	LIBOR	CDS Spread					
Date	12 Month	AAA	AA	A	BBB	BB	B
20140430	0.5490%	0.0938%	0.1670%	0.3881%	0.5722%	1.7952%	4.1154%
20140429	0.5490%	0.1105%	0.1679%	0.3985%	0.5913%	1.7995%	4.1502%
20140428	0.5495%	0.1113%	0.1685%	0.3992%	0.5894%	1.9206%	4.2556%
20140425	0.5495%	0.1113%	0.1673%	0.3826%	0.5697%	1.8139%	4.2349%
20140424	0.5495%	0.0968%	0.1676%	0.3807%	0.5806%	1.5016%	4.2173%
20140423	0.5483%	0.1111%	0.1686%	0.4037%	0.5774%	1.7221%	4.3761%
…	…	…	…	…	…	…	…
20090507	1.7813%	0.3075%	0.9621%	1.6332%	3.4747%	8.4460%	28.0827%
20090506	1.8200%	0.3599%	1.1833%	1.8355%	3.6819%	8.8693%	26.2862%
20090505	1.8589%	0.3695%	1.1359%	1.8929%	3.8222%	9.1412%	26.5660%
20090504	1.8644%	0.4343%	1.2053%	2.0017%	3.8649%	9.3918%	26.4036%
20090501	1.8644%	0.4366%	1.1579%	2.0539%	3.9778%	8.8810%	26.9790%
variance	0.0007%	0.0001%	0.0005%	0.0007%	0.0032%	0.0168%	0.2053%
mean	0.909%	0.2476%	0.5264%	0.7041%	1.0387%	2.7020%	7.4352%

And we know the conditional variance of the interest rate at any time *t* is

22 $$\begin{array}{*{20}l} Variance[r_{t}|r_{0}]=r_{0}\frac{\sigma_{1}^{2}}{a_{1}}\left(e^{-a_{1}t}-e^{-2a_{1}t}\right)+\frac{b_{1}\sigma_{1}^{2}}{2a_{1}}\left(1-e^{-a_{1}t}\right)^{2}. \end{array} $$

For sufficient large *t*, this variance will turn into a long term variance and *r*_0_ has almost no effect. Let *t*=100. It is reasonable to assume that the long term variance equals to the variance of the 12 month LIBOR rate. Then we can have

$$\sigma_{1}=\sqrt{\frac{Variance(\text{data})}{\frac{b_{1}}{2a_{1}}(1-e^{-100a_{1}})^{2}}} $$

and

$$Variance(\text{data})=\frac{\sum_{i=1}^{N}(r_{i}-\bar{r})^{2}}{N-1}, $$

where $\bar {r}=\frac {1}{N}\sum ^{N}_{i=1}r_{i}.$ Assuming *a*_1_=1, we have *σ*_1_=0.038060013. These estimated parameters of *b*_1_,*σ*_1_ and *a*_1_ have been given in Table 2.

**Table 2:** Interest rate model parameters estimations

*r*_0_	*b*_1_	*σ*_1_	*a*_1_
0.5490%	0.909%	0.038060013	1

For the hazard rate, a common model applied in industry is the CDS spread approach. This model assumes

23 $$\begin{array}{*{20}l} \lambda(t)=\frac{\text{spread}(t)}{1-R}, \end{array} $$

where *R* is recovery rate and is normally assumed to be 0.4.

We used the USD Financial sector 12 Month CDS spread data from May 1, 2009 to April 30, 2014. Partial data of these CDS spreads is given in Table 1. Applying the model in (23), we used the rate on April 30, 2014 to approximate the initial hazard rate. The long term average is estimated by

$$b_{2}=\frac{mean(\text{data})}{1-R} $$

where

$$mean(\text{data})= \frac{1}{N}\sum^{N}_{i=1}{spread}_{i}. $$

And we have the same formula for conditional variance of the hazard rate at any time *t* as (22),

24 $$\begin{array}{*{20}l} Variance[\lambda_{t}|\lambda_{0}]=\lambda_{0}\frac{\sigma_{2}^{2}}{a_{2}}\left(e^{-a_{2}t}-e^{-2a_{2}t}\right)+ \frac{b_{2}\sigma_{2}^{2}}{2a_{2}}\left(1-e^{-a_{2}t}\right)^{2}. \end{array} $$

Since the relationship between the hazard rate and spread in (23) exists, it is reasonable to assume the variance of spread equal to the variance of the hazard rate. Then we have

$$\sigma_{2}=\sqrt{\frac{Variance(\text{data})}{\frac{b_{2}}{2a_{2}}(1-e^{-100a_{2}})^{2}}} $$

and

$$Variance(\text{data})=\frac{\sum_{i=1}^{N}\left({spread}_{i}-\frac{1}{N}\sum^{N}_{i=1}{spread}_{i}\right)^{2}}{N-1}. $$

Assume *a*_2_=1, we can then get *σ*_2_. All these parameters for different ratings are shown in Table 3.

**Table 3:** Hazard Rate Model Parameters estimations

	*λ*_0_	*b*_2_	*σ*_2_	*a*_2_
AAA	0.15633%	0.4127%	0.020113992	1
AA	0.27833%	0.8774%	0.032584086	1
A	0.64683%	1.1736%	0.035502957	1
BBB	0.95367%	1.7312%	0.060824805	1
BB	2.99200%	4.5034%	0.086378396	1
B	6.85900%	12.3920%	0.182026115	1

The rating A is normally used as a testing grade. So we used the parameters for rating A here, i.e., *λ*_0_=0.64683*%*,*b*_2_=1.1736*%*,*σ*_2_=0.035502957,*a*_2_=1. The other parameters are set as follows, maturity *T* is set to be 5 years. The fixed rate *K* for original swap is 0.909%, *ρ*=0.2 and the notional value is $250,000,000.

### Default-free replacement contracts

We use a 100 ×100 grid for the interest rate and hazard rate. The number of time steps is 600 over a 5 year period. The price for CCIRS is $2,236.22. This computation takes less than 1.9 seconds and the result is close to the result obtained by Monte Carlo simulation. When we increase the number of time steps to 2000, which is the same as the number of the Monte-Carlo simulations, the total computational time increases to 3.62 seconds and the price is $2,235.02, which suggests that the time step is sufficiently acceptable for the spatial grid chosen. We have also obtained the result by assuming a constant hazard rate, which is obtained by solving the reduced PDE, with a 100 grid points in *r* and 600 time steps over 5 years. The price for a constant hazard rate is $1,201.53, which is quite different from $2,235.02, obtained by solving the PDE with a stochastic hazard rate.

Table 4 shows the comparison of results obtained by using the PDE method and the Monte Carlo simulation. The results are consistent with each other.

**Table 4:** Comparison of results obtained using PDE and Monte-Carlo methods for one default case (maturity=5, *K*=0.00909, 1 million simulation paths)

	PDE(ADI)	PDE(ADI)	Monte Carlo
	time steps 600	time steps 2000	99.9% confidence interval
Price	$ 2,236.22	$ 2,235.02	$ [2173.94, 2235.24]
Time (seconds)	1.19	3.62	648

**Fig. 1:**
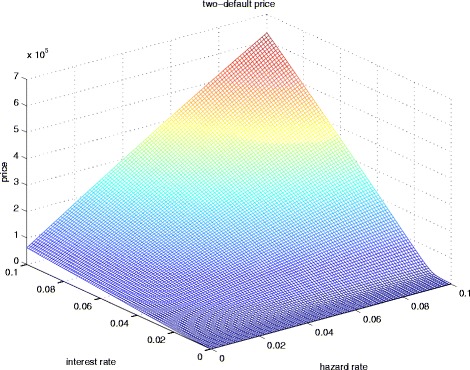
CCIRS price as a function of interest and hazard rates

In addition to the savings in computational time, the PDE approach also generates the price of CCIRS for the entire range of interest and hazard rates, as shown in Fig. 1. It can be seen that the price is in general an increasing function of the interest and hazard rates, since higher hazard rates mean higher probability of default.

### Defautable replacement contracts

We used the same parameters given in the previous section. In the Monte-Carlo simulations, we ran 1,000,000 realizations and partitioned the time to maturity (ie., 5 years) into 2,000 equal time intervals. The computational time is 660 seconds and the price is $2,223.51.

The PDEs are solved based on a 600×100×100 grid over the range of hazard rate. The computational time is 4.07 seconds, due to the fact that we need to solve four PDEs. The CCIRS price is $2,263.50. These results are consistent to the ones from the Monte-Carlo simulation and the detail is given in Table 5.

**Table 5:** Comparison of results using PDEs and Monte-Carlo methods for the two default case

	PDE(ADI)	PDE(ADI)	Monte Carlo 99.9% confidence interval
	time steps 600	time steps 2000	time steps 2000
Price	$ 2,264.26	$ 2,263.50	$ [2202.96, 2264.08]
Time (seconds)	4.07	14.68	660

(maturity=5, *K*=0.00909, 1 million simulation paths)

**Fig. 2:**
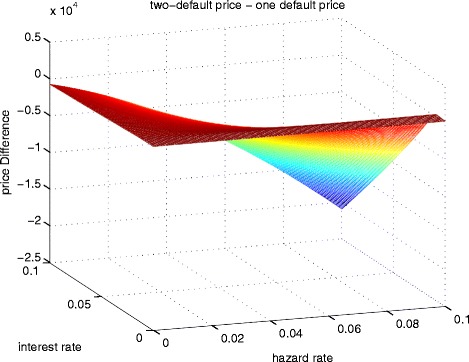
Difference of two-default and one-default price

The PDE technique can also provide the solution on any point of the grid. We have chosen a simulated path for *r* and *λ*. Using the same time for calculating the price of CCIRS with 2-default, the results are shown in Table 6. The comparisons between the price computed based on default-free replacement contract (one-default) and defaultable replacement contract (two-default) are presented in Tables 7,8 and 9, and illustrated in Fig. 2. It can be seen that the difference between the two prices can be significant under certain conditions, especially for counterparties with lower credit ratings.

**Table 6:** Price of CCIRS on different annual node (maturity =5, *K* =0.00909)

Year	*r*(*t*)	*λ*(*t*)	Price
0	0.5490%	0.6468%	2,264.26
1	0.5301%	0.5383%	1,293.70
2	0.6261%	0.6664%	705.00
3	0.7932%	1.0683%	401.82
4	0.9933%	0.8382%	146.92

**Table 7:** Comparison of the one-default and two-default cases (# of time step=2000, maturity=5, *K*=0.00909)

	One-default	Two-default
Price	$ 2,235.02	$ 2,263.50

**Table 8:** Comparison of the one-default and two-default cases under various correlation (# of time step =600, maturity =5, *K* =0.01)

*ρ*	One-default	Two-default
0	2,075	2,100
0.1	2,155	2,182
0.2	2,236	2,264
0.3	2,319	2,349
0.4	2,404	2,435
0.5	2,490	2,522
0.6	2,578	2,611
0.7	2,668	2,702
0.8	2,759	2,794

**Table 9:** Comparison of the one-default and two-default cases over a range of credit ratings

Rating	One-default	Two-default
AAA	303.76	310.24
AA	632.60	661.51
A	822.47	875.13
BBB	1,215.01	1,330.24
BB	2,548.34	3,185.29
B	4,224.85	6,964.76

(maturity =10, *K* =0.01)

## Conclusion

In this paper, we have investigated a model that allows for multiple defaults of counterparties in Credit Valuation Adjustment, which received more attention after the 2008 financial crisis.

Previous work on CVA has not assessed the default risk by the replacement contract. By extending the pricing model that allows a second default, we derived and numerically solved a system of four coupled PDEs. Our PDE approach is much more efficient than the Monte Carlo simulation. Our results suggest that subsequent defaults cannot be ignored for firms with lower credit ratings.

In principle, the counterparty of the replacement contract can also default. By restricting to the case of two-defaults, we potentially also can overestimate or underestimate the true risk. This paper laid the foundation for more general cases. We are currently exploring this issue and the results will be reported in a subsequent paper.
